# Plasma DCLK1 is a marker of hepatocellular carcinoma (HCC): Targeting DCLK1 prevents HCC tumor xenograft growth via a microRNA-dependent mechanism

**DOI:** 10.18632/oncotarget.5808

**Published:** 2015-10-16

**Authors:** Sripathi M. Sureban, Mohammad F. Madhoun, Randal May, Dongfeng Qu, Naushad Ali, Javid Fazili, Nathaniel Weygant, Parthasarathy Chandrakesan, Kai Ding, Stanley A. Lightfoot, Courtney W. Houchen

**Affiliations:** ^1^ Department of Medicine, The University of Oklahoma Health Sciences Center, Oklahoma City, OK 73104, USA; ^2^ Department of Biostatistics & Epidemiology, The University of Oklahoma Health Sciences Center, Oklahoma City, OK 73104, USA; ^3^ Department of Pathology, The University of Oklahoma Health Sciences Center, Oklahoma City, OK 73104, USA; ^4^ Department of Veterans Affairs Medical Center, Oklahoma City, OK 73104, USA; ^5^ The Peggy and Charles Stephenson Cancer Center, Oklahoma City, OK 73104, USA; ^6^ COARE Biotechnology Inc., Oklahoma City, OK 73104, USA

**Keywords:** circulating DCLK1, biomarker, miRNA, cirrhosis, HCC

## Abstract

Tumor stem cell marker Doublecortin-like kinase1 (DCLK1) is upregulated in several solid tumors. The role of DCLK1 in hepatocellular carcinoma (HCC) is unclear. We immunostained tissues from human livers with HCC, cirrhosis controls (CC), and non-cirrhosis controls (NCC) for DCLK1. Western blot and ELISA analyses for DCLK1 were performed with stored plasma samples. We observed increased immunoreactive DCLK1 in epithelia and stroma in HCC and CCs compared with NCCs, and observed a marked increase in plasma DCLK1 from patients with HCC compared with CC and NCC. Analysis of the Cancer Genome Atlas’ HCC dataset revealed that DCLK1 is overexpressed in HCC tumors relative to adjacent normal tissues. High DCLK1-expressing cells had more epithelial-mesenchymal transition (EMT). Various tumor suppressor miRNAs were also downregulated in HCC tumors. We evaluated the effects of DCLK1 knockdown on Huh7.5-derived tumor xenograft growth. This was associated with growth arrest and a marked downregulation of cMYC, and EMT transcription factors ZEB1, ZEB2, SNAIL, and SLUG *via let-7a* and *miR-200* miRNA-dependent mechanisms. Furthermore, upregulation of *miR-143/145*, a corresponding decrease in pluripotency factors OCT4, NANOG, KLF4, and LIN28, and a reduction of *let-7a*, *miR-143/145,* and *miR-200*-specific luciferase activity was observed. These findings suggest that the detection of elevated plasma DCLK1 may provide a cost-effective, less invasive tool for confirmation of clinical signs of cirrhosis, and a potential companion diagnostic marker for patients with cirrhosis and HCC. Our results support evaluating DCLK1 as a biomarker for detection and as a therapeutic target for eradicating HCC.

## INTRODUCTION

Hepatocellular carcinoma (HCC) is the fifth most common cancer worldwide [1]. Over 80% of HCC is associated with liver cirrhosis and hepatitis [2]. Chronic viral infections are major risk factors [3]. HCC is an aggressive tumor with poor prognosis; median survival after diagnosis ranges from ~6 to 20 months [4]. Liver transplantation or resection is the first line of treatment [5]. However, only 25% of patients are eligible for curative resection. Overall survival is dismal for ineligible patients [6].

Tissue stem cells are long-lived rare cells that acquire the ability to self-renew. When mutated, these cells can act as tumor stem cells (TSCs) or cancer stem cells (CSCs) [7]. Several proteins have emerged as potential markers for the identification of TSCs in HCC: CD133, CD90, CD24, CD44, CD13, oval cell marker 6 (OV6), side population (SP), Aldehyde Dehydrogenase (ALDH) activity, and the epithelial cell adhesion molecule (EpCAM) [8, 9]. Recently, CD133+ cells that were isolated from human HCC tissues and sequentially passaged were shown to undergo epithelial-mesenchymal transition (EMT), and demonstrated aggressive tumor growth and metastasis [10]. These findings suggest that EMT and metastasis are linked and may represent a unique characteristic of HCC TSCs.

Pluripotency/reprogramming factors OCT3/4, SOX2, KLF4, cMYC, and NANOG were shown to play roles in HCC and CSC development and maintenance [8, 11]. Furthermore, MYC-driven hepatic tumors were reported to contain a subset of cells with CSC (SP+) traits [11]. NANOG was also demonstrated to play an important role in the self-renewal of CSCs expressing CD24 or CD133 [8, 12].

Several researchers suggested that the presence of liver CSCs in resected specimens is associated with poor prognosis in HCC. Stemness was identified as a predictive marker of HCC and intrahepatic cholangiocarcinoma prognosis [13]. CSCs are reported to be highly invasive and metastatic, and can be isolated/detected in peripheral blood mononuclear cells as circulating tumor cells. Thus, CSCs may provide diagnostic or prognostic information [8]. Taken together, these data suggest that EMT and pluripotency factors may function as oncogenes to generate liver CSCs, and CSC markers can be used as biomarkers for HCC detection.

The doublecortin-like kinase 1 (DCLK1), a microtubule-associated kinase, is a putative marker of the intestine and pancreas. Gordon *et al*. (2008) [14] identified Dclk1 as a gastric epithelial progenitor or gastric stem cell. We showed that Dclk1 marks a subset of quiescent cells in the normal intestine and is upregulated in *Apc^Min/+^* adenomas [15]. Researchers demonstrated that Dclk1 marks TSCs that continuously produce tumor progeny in the intestinal polyps of *Apc^Min/+^* mice, and suggested that Dclk1 marks the cell of origin in an *Apc^Min/+^* model of intestinal tumorigenesis [16]. We demonstrated that chronic hepatitis C infection predisposes cells to acquire CSC-like traits while inducing DCLK1 and hepatic progenitor and stem cell-related factors [17, 18]. Numerous reports have established that DCLK1 regulates tumor suppressor miRNAs that play key roles in tumor initiation, progression, and metastasis [19–23]. Targeting DCLK1 arrested colorectal and pancreatic tumor xenograft growth via inhibition of EMT, pluripotency, and critical oncogenic pathways [19–23].

In the present study, we found increased expression of DCLK1 in plasma and epithelial and stromal compartments of tissues with cirrhosis and HCC compared with non-cirrhotic controls (NCCs). Furthermore, we observed a statistically significant increase in DCLK1 expression in HCC compared with controls. Treatment of Huh7.5 human hepatoma cell-derived tumor xenografts with DCLK1-specific siRNA produced tumor growth arrest, DCLK1 downregulation, and increased expression of tumor suppressor miRNAs *let-7a*, *miR-200*, and *miR-143/145*. A subsequent inhibition of factors that promote tumorigenesis, including cMYC and pluripotency and EMT factors, was observed. These results indicate that DCLK1 can be used as a biomarker for the detection of HCC and may be a candidate for developing targeted therapeutics to eradicate HCC.

## RESULTS

### DCLK1 is upregulated in HCC and cirrhotic controls compared with non-cirrhotic controls

**Figure 1:**
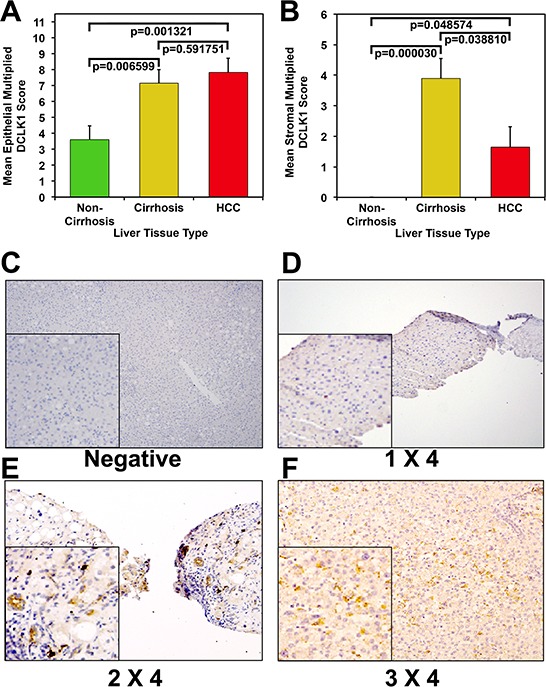
Increased DCLK1 protein expression in human hepatocellular carcinoma and cirrhotic controls compared with non-cirrhotic controls **A.** Mean epithelial multiplied DCLK1 score among the three groups. **B.** Mean stromal multiplied DCLK1 score among the three groups. Immunohistochemical staining for DCLK1. **C.** Negative; **D.** representative image of tissue with DCLK1 staining (brown) intensity score of 1 and tissue involvement score 4 (composite scoring 1 × 4); **E.** representative image of tissue with DCLK1 staining (brown) intensity score of 2 and tissue involvement score of 4 (2 × 4), and **F.** representative image of tissue with DCLK1 staining (brown) intensity score of 3 and tissue involvement score of 4 (3 × 4).

Twenty-three NCC cases, 22 CCs, and 23 HCCs surgical specimens were included in the histopathology analysis. Differences in mean epithelial multiplied scores were statistically significant between CCs and NCCs (7.15 vs. 3.6, *p* = 0.0006599), and between HCCs and NCCs (7.82 vs. 3.6, *p* = 0.0001321), but not between HCCs and CCs (7.82 vs. 7.15, *p* = 0.591751; Figure 1A). We assessed the results of stromal staining for DCLK1 in 17 HCCs, 19 CCs, and 20 NCCs. The mean stromal multiplied score was significantly higher in CCs than in NCCs (3.89 vs. 0, *p* = 0.000030), compared with HCCs (3.89 vs. 1.64, *p* = 0.038810), and was significant when HCCs were compared with NCCs (1.64 vs. 0, *p* = 0.048574; Figure 1B). Figure panels 1C–1F show representative images of DCLK1 immunostaining.

We examined the overall clinical characteristics of 23 HCCs in more detail in relation to DCLK1 staining. HCCs were considered DCLK1-positive if the composite multiplied score was ≥ 3 (*n* = 19). Four HCCs were considered DCLK1-negative. Sixty-one percent (14/23) were positive for HCV. Eighteen percent (4/22) had early stage disease (stage I or II based on TNM staging). The mean age was 62 ± 13.8 years. No significant differences in clinical predictor variables were identified between the HCCs by DCLK1 positivity.

There was a trend toward higher AFP levels among DCLK1-positive HCCs, in which the median AFP was 167, compared with 6 in the DCLK1-negative group (*p* = 0.07). DCLK1-positive cases also tended to have more than one lesion compared with DCLK1-negative cases (74% vs. 25%, *p* = 0.06), and were more likely have vascular invasions on histopathology (28% vs. 0%, *p* = 0.54). Despite these trends, differences were not statistically significant (Supplementary Table S1). The simple Kappa coefficient for intra-observer agreement was 0.67 (95% CI [0.41–0.93]) for the multiplied epithelial score, suggesting excellent agreement. The same was true when agreement was tested for the amount scoring by itself (*K* = 0.63; 95% CI [0.25–1]), and was excellent when tested for the intensity scoring by itself (*K* = 0.81; 95% CI [0.58–1]).

### DCLK1 protein is elevated in the plasma of patients with HCC

**Figure 2:**
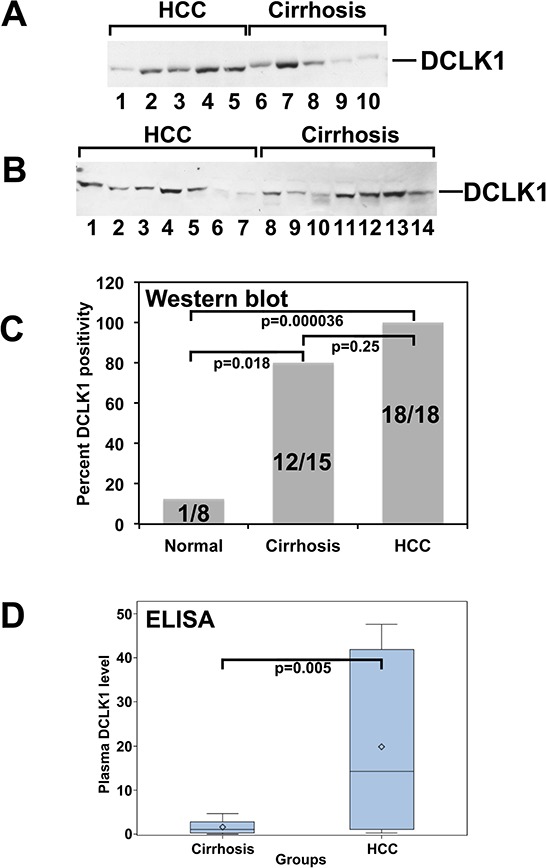
DCK1 protein levels are elevated in the plasma of patients with hepatocellular carcinoma. DCLK1 protein was detected and estimated using western blots and ELISA, respectively **A.** and **B.** Western blot of representative plasma samples for DCLK1. **C.** Bar graph demonstrating the percent of samples positive for DCLK1. **D.** DCLK1 protein levels in plasma estimated by ELISA. Bar graph demonstrates the DCLK1 levels in cirrhosis and HCC patients.

Eighteen HCCs, 15 CCs, and 8 NCCs were included in the Western blot plasma analysis. DCLK1 was detectable in the plasma of all HCCs, 12/15 (80%) CCs, and 1/8 (12%) of NCCs (Figure 2A–2C). There exists a significant difference between HCCs, CCs and NCCs (*p* < 0.0001, overall χ2 test). The difference between HCCs and NCCs was statistically significant (Bonferroni-adjusted *p* = 0.000036).

In the ELISA study, 18 HCCs and 15 CCs were utilized. In CCs, we observed an average DCLK1 level of 1.61 ng/mL, with a median of 1.11 (min 0.03 and max 4.70). In contrast, DCLK1 levels were significantly elevated in HCCs, with a mean of 19.82 and a median of 14.22 (min 0.27 and max 47.59), *p* = 0.005 (Figure 2D). The analysis was performed using the Wilcoxon rank-sum test.

### DCLK1 is overexpressed in HCCs: DCLK1^High^-expressing HCCs have more EMT

**Figure 3:**
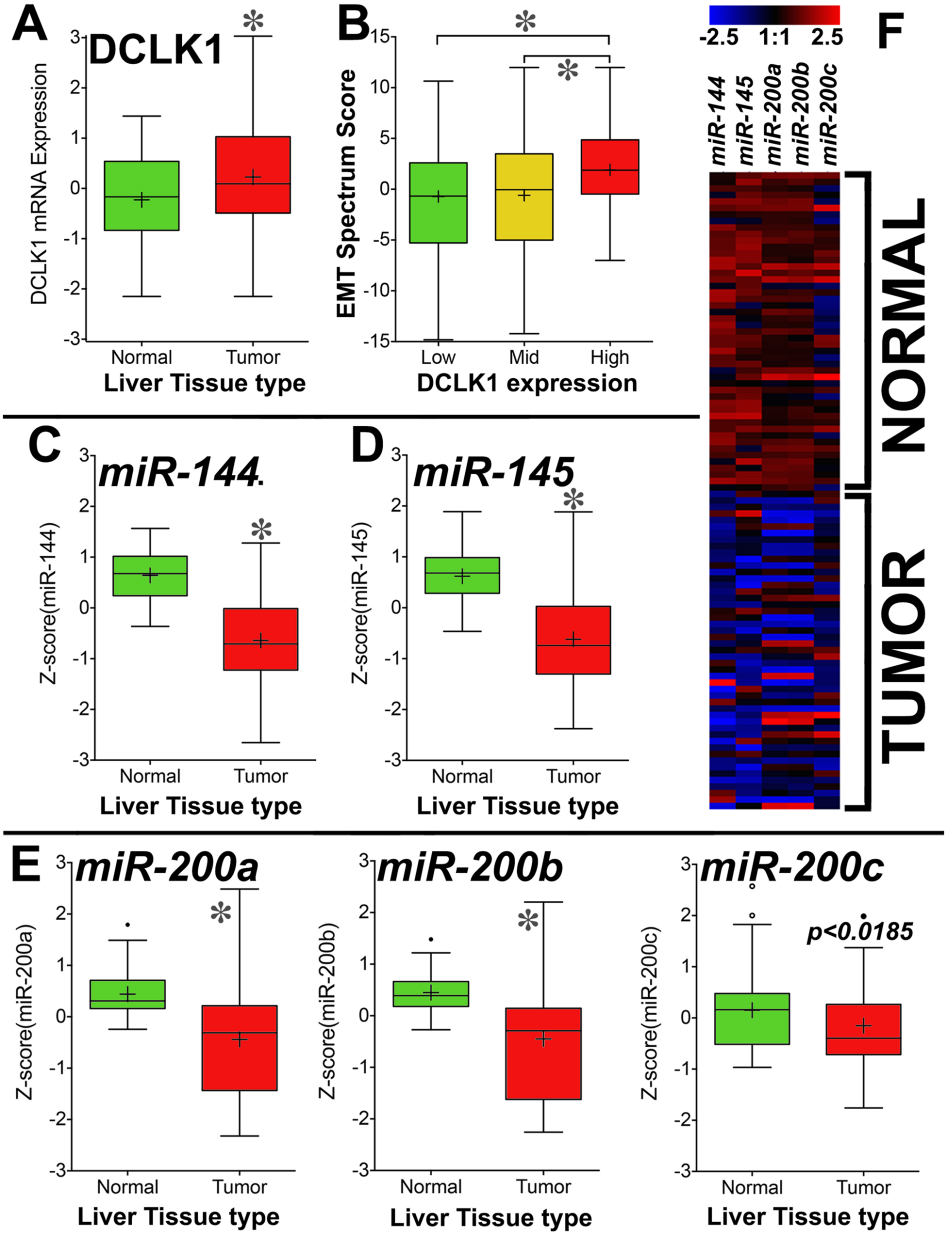
DCLK1 mRNA is overexpressed and tumor suppressor miRNAs are downregulated in TCGA's Liver Hepatocellular Carcinoma (LIHC) RNA-seq dataset **A.** DCLK1 mRNA is significantly overexpressed in HCC patient tumors compared with matched adjacent normal tissue (*p* < 0.0001). **B.** Clinical outcome EMT spectrum scores were calculated for DCLK1^Low^-, DCLK1^Mid^-, and DCLK1^High^- expressing tumors. We observed significantly higher EMT scores in DCLK1^High^- and DCLK1^Mid^- expressing tumors than in DCLK1^Low^-expressing tumors (*p* < 0.0001). Tumor suppressor miRNAs *miR-144* **C.** *miR-145* **D.** and *miR-200a, b, c* **E.** were significantly (*p* < 0.0001, except for *miR-200c*) downregulated in HCC tumors compared with adjacent normal tissue. **F.** Heat map demonstrating the expression of miRNAs in HCC tumors and adjacent normal tissue (*n* = 49 each). Values in the bar graphs are given as average ± *SEM*. Asterisks denote statistically significant differences (*p < 0.0001).

Analysis of The Cancer Genome Atlas’ (TCGA) Liver Hepatocellular Carcinoma (LIHC) dataset revealed that DCLK1 is overexpressed in HCC tumors (*n* = 373) compared with adjacent normal tissue (*n* = 50) (Figure 3A). DCLK1^Mid^- and DCLK1^High^-expressing tissues had significantly higher EMT spectrum scores than DCLK1^Low^-expressing tissues (Figure 3B).

We analyzed the expression of various tumor suppressor miRNAs (*miR-144, miR-145,* and *miR-200a, b, c*) that are regulated by DCLK1. We observed significant downregulation of *miR-144* (Figure 3C and 3F), *miR-145* (Figure 3D and 3F), and *miR-200a, b, c* (Figure 3E and 3F) in HCC tumors compared with adjacent normal tissue. These data indicate that DCLK1 overexpression in HCC may induce EMT and downregulate tumor suppressor miRNAs. This supports our hypothesis that DCLK1+ cells in HCC may undergo EMT and can be detected in the bloodstream as a biomarker for cirrhosis and HCC.

### Inhibition of DCLK1 results in liver cancer tumor xenograft growth arrest

**Figure 4:**
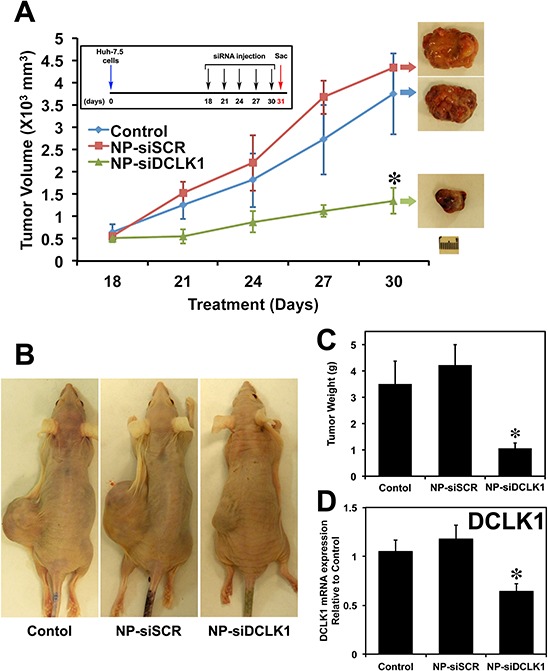
siRNA-mediated knockdown of DCLK1 results in human liver tumor xenograft growth arrest **A.** Huh7.5 human liver cancer cells were subcutaneously injected into the flanks of athymic nude mice to generate tumors. At day 18, PLGA NP encapsulated siRNAs (NP-siDCLK1 and NP-siSCR) or NPs alone (Control) (*n* = 4 animals per group) were injected via i.p., followed by injections every third day. After 5 injections, tumors were excised on day 31 and are shown above. Tumor volume was measured every 3 days. **B.** Representative photograph of tumor-bearing mice from each group are shown. **C.** Average weight of the excised tumors. **D.** siRNA-mediated knockdown of DCLK1 results in decreased expression of DCLK1 mRNA in tumor xenografts. Values are given as average ± *SEM*. Asterisks denote statistically significant differences (*p < 0.01) compared with Control (NP alone).

To demonstrate a regulatory role of DCLK1 in HCC tumorigenesis, we generated tumor xenografts and examined the effects of treatment with siRNAs. Figure 4A shows the tumor volumes measured on the days of injection (*n* = 4 animals per group). There were no significant differences in the tumor volumes between NPs alone (Control) and NP-siSCR-treated tumors (NPs were administered via i.p.). Administration of NP-siDCLK1 produced a significant (~75%) reduction (*p* < 0.01) in tumor volume compared with the Control and NP-siSCR-treated tumors (Figure 4A, 4B). NP-siDCLK1-treated tumors weighed significantly less than control and NP-siSCR-treated tumors (*p* < 0.01; Figure 4C). These data indicate that DCLK1 inhibition results in Huh7.5 tumor xenograft growth arrest.

### DCLK1 negatively regulates miRNA *let-7a* and affects downstream oncogenic signaling

cMYC is reported to be overexpressed in ~70% of viral and alcohol-related chronic liver diseases and HCC, signifying a more advanced and aggressive phenotype of HCC, indicating that cMYC plays a critical role in the pathogenesis of HCC or liver cancer. Little research has pursued the development of cMYC as a target. Based on previous publications, we suspect that DCLK1 regulates the oncogene cMYC.

**Figure 5:**
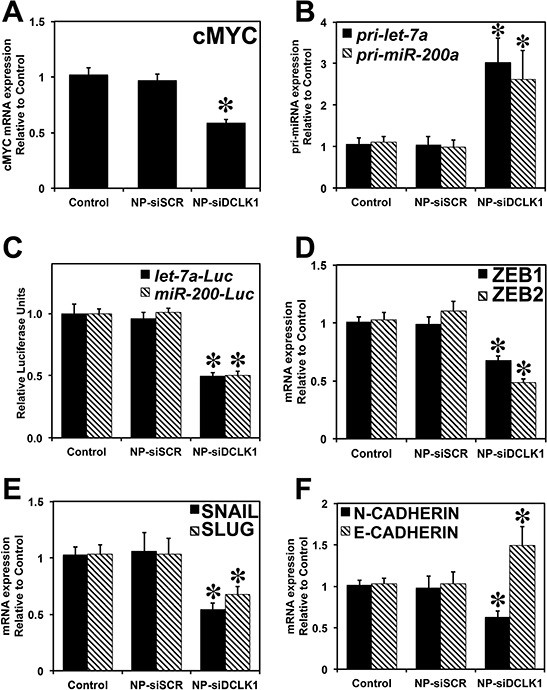
Knockdown of DCLK1 results in inhibition of cMYC via *let-7a* and EMT via *miR-200* **A.** Decreased expression of cMYC mRNA in NP-siDCLK1-treated tumors. **B.** Increased expression of *pri-let-7a* and *pri-miR-200a* miRNAs following the knockdown of DCKL1 in tumor xenografts. **C.** siRNA-mediated knockdown of DCLK1 resulted in a decrease in *miR-let-7a* and *miR-200*-dependent luciferase activity was observed in Huh7.5 cells. Tumor xenografts treated with NPsiDCLK1 demonstrated a downregulation of EMT transcription factors ZEB1 and ZEB2 mRNA **D.** decreased SNAIL and SLUG mRNA expression **E.** decreased N-CADHERIN mRNA and increased expression of E-CADHERIN **F.** Values in the bar graphs are given as average ± *SEM*. Asterisks denote statistically significant differences (*p < 0.01) compared with Control (NP alone).

We evaluated the expression of levels of DCLK1 and cMYC in tumor xenografts treated with siRNAs. A nearly 40% reduction in DCLK1 mRNA (*p* < 0.01) was observed in tumors treated with NP-siDCLK1 compared with control and NP-siSCR-treated tumors, indicating effective administration of the DCLK1 siRNA that ultimately reached the tumors (Figure 4D). We observed nearly a 50% downregulation of cMYC mRNA in NP-siDCLK1-treated tumors compared with control and NP-siSCR-treated tumors (Figure 5A). These findings demonstrate that DCLK1 knockdown results in downregulation of cMYC mRNA in tumor xenografts.

Earlier reports suggested that cMYC is a target of miRNA *let-7a*. We examined whether DCLK1 regulates cMYC *via* a *let-7a* miRNA-dependent mechanism. We observed a significant (*p* < 0.01) upregulation of *let-7a* pri-miRNA (>2.5-fold) in tumors treated with NP-siDCLK1 compared with control and NP-siSCR-treated tumors (Figure 5B), indicating that DCLK1 knockdown induces miRNA *let-7a* in tumor xenografts. To demonstrate whether DCLK1 negatively regulates *let-7a* and its downstream targets post-transcriptionally, we transfected Huh7.5 cells with plasmid containing firefly luciferase gene with complimentary miRNA *let-7a* binding sites at the 3′UTR. Upon transfection, the cells were treated with either NP-alone, NP-siSCR, or NP-siDCLK1, and were subjected to luciferase activity measurement. A significant (*p* < 0.01) downregulation (~50%) of *let-7a*-dependent luciferase activity was observed in the cells treated with NP-siDCLK1 compared with control and NP-siSCR-treated cells (Figure 5C). These data indicate that DCLK1 negatively regulates *let-7a*, and DCLK1 knockdown downregulates cMYC via a *let-7a*-dependent mechanism.

### Inhibition of DCLK1 results in downregulation of EMT-related genes via *miR-200*

EMT plays a crucial role in the metastatic spread of liver cancer and causes cells and proteins to shed into the circulation. We predicted that DCLK1 plays a regulatory role in EMT, and that DCLK1 negatively regulates *miR-200*. Following DCLK1 knockdown, we observed a significant (*p* < 0.01) upregulation of *miR-200a* pri-miRNA (>2.5-fold) in tumors treated with NP-siDCLK1 compared with control and NP-siSCR-treated tumors (Figure 5B). To demonstrate that DCLK1 negatively regulates *miR-200*, we transfected the Huh7.5 cells with plasmid containing luciferase gene under the control of 3′UTR containing *miR-200* binding site. Following DCLK1 knockdown, a significant downregulation (>50%, *p* < 0.01) in *miR-200*-dependent luciferase activity was observed (Figure 5C). These results show that DCLK1 negatively regulates tumor and EMT suppressor miRNA *miR-200* in liver cancer, and DCLK1 affects *miR-200* downstream targets. In xenografts treated with NP-siDCLK1, we observed a reduction of *miR-200* downstream targets ZEB1, ZEB2, (Figure 5D), SNAIL, and SLUG (Figure 5E), compared with controls and tumors treated with NP-siSCR. We also observed significant downregulation of N-CADHERIN and E-CADHERIN rescue (upregulated nearly 1.5-fold) in tumors treated with NP-siDCLK1 (Figure 5F). These data indicate that DCLK1 plays a crucial role in promoting EMT. This process may be responsible for detection of DCLK1 in the circulation.

### DCLK1 controls pluripotency factors expression *via* post-transcriptional regulation of *miR-143/145* in liver cancer

**Figure 6:**
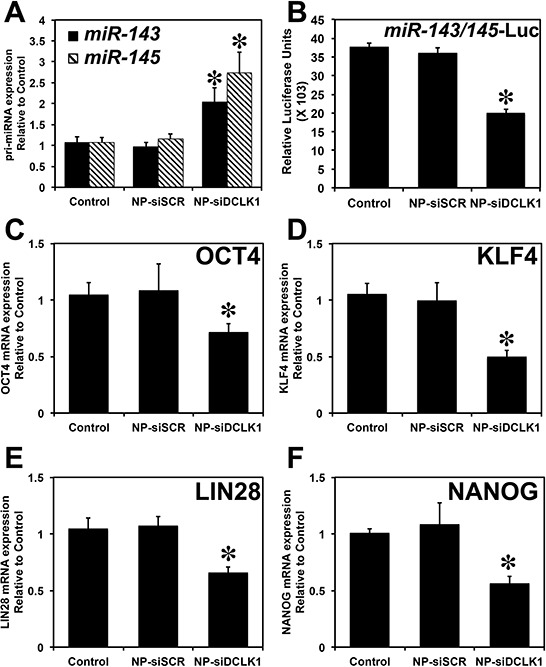
DCLK1 regulates pluripotency via post-transcriptional regulation of *miR-143/145* **A.** Following the knockdown of DCLK1 in Huh7.5 tumor xenografts, RT-PCR revealed a significant upregulation of *miR-143* and *miR-145* miRNA. **B.** A decrease in luciferase activity (luciferase units) following transfection with plasmid-encoding luciferase containing the *miR-143/145* binding site was observed following the knockdown of DCLK1 in Huh7.5 human liver cancer cells. siRNA-mediated knockdown of DCLK1 resulted in downregulation of pluripotency factors: OCT4 mRNA **C.** KLF4 mRNA **D.** LIN28 **E.** and NANOG **F.** Values in the bar graphs are given as average ± *SEM*. Asterisks denote statistically significant differences (*p < 0.01) compared with Control (NP alone).

We previously demonstrated that DCLK1 post-transcriptionally regulates pluripotency factors *via miR-143/145* in pancreatic cancer. Here, we investigated whether DCLK1 negatively regulates *miR-143/145* in liver cancer. mRNA isolated from siRNA-treated tumors were analyzed for *miR-143* and *miR-145*. We observed a significant (*p* < 0.01) upregulation (>2-fold) in *miR-143* and *miR-145* expression in tumors treated with NP-siDCLK1 compared with control and NP-siSCR-treated tumors (Figure 6A). Based on earlier reports that DCLK1 negatively regulates tumor suppressor miRNAs post-transcriptionally, we performed luciferase reporter gene-based assays. Huh7.5 cells were transfected with a vector containing firefly luciferase gene with complimentary *miR-143/145* binding sites at the 3′UTR. Upon transfection, the cells were treated with NP-alone, NP-siSCR, or NP-siDCLK1, and were subjected to luciferase activity measurement. A significant (*p* < 0.01) downregulation (~50%) of *miR-143/45*-dependent luciferase activity was observed in NP-siDCLK-treated cells compared with control and NP-siSCR-treated cells (Figure 6B). These results show that DCLK1 negatively regulates *miR-143/145*, and DCLK1 knockdown may downregulate *miR-143/154* downstream pluripotency transcription factors.

We next evaluated the expression of pluripotency factors OCT4, KLF4, LIN28, and NANOG. DCLK1 knockdown resulted in decreased expression of OCT4 (>30%, Figure 6C), KLF4 (>45%, Figure 6D), LIN28 (>40%, Figure 6E), and NANOG (>50%, Figure 6F) in tumor xenografts. These differences were statistically significant (*p* < 0.01) when compared with control and NP-siSCR-treated tumors. These findings revealed that DCLK1 regulates pluripotency factors *via* an *miR-143/145*-dependent mechanism in liver cancer.

## DISCUSSION

Here, we demonstrated increased DCLK1 expression in the epithelial and stromal compartments of tissues with cirrhosis and HCC. Furthermore, we observed a statistically significant increase in plasma DCLK1 expression in HCC compared with controls. These data indicate that DCLK1 can be used as a biomarker for cirrhosis and HCC. We also found that treating Huh7.5 human hepatoma cell-derived tumor xenografts with DCLK1-specific siRNA resulted in tumor growth arrest, downregulation of DCLK1, and increased expression of tumor suppressor miRNAs *let-7a*, *miR-200*, and *miR-143/145*. A subsequent inhibition of factors promoting cMYC, pluripotency, and EMT was observed. Thus, DCLK1 may be a candidate for the development of therapeutics to eradicate HCC.

These data suggest that DCLK1 marks certain stem-like cells that may have the potential for tumor initiation, and which might harbor HCC CSCs in cirrhotic livers. One possible explanation is that circulating bone marrow-derived mesenchymal cells, along with other resident progenitor cells in the liver, may be recruited into inflamed or infected livers to facilitate tissue repair. In chronic liver disease, these stem-like cells can increase with disease severity and may undergo a phenotypic shift and further differentiate into tumor cells, which then progress to HCC or differentiate into hepatic-lineage cells to support liver regeneration [8, 24]. Our findings support the hypothesis that hepatic stem cells may be involved in hepatocarcinogenesis.

DCLK1 protein was detected in the plasma of all patients with HCC and in 80% of cirrhosis controls, consistent with our archived histopathology study, even though the studies were conducted with samples from different repositories. We are the first to demonstrate detectable DCLK1 in the plasma of patients with cirrhosis and HCC. Although the origin of the DCLK1 protein is unclear, studies are underway to determine whether it represents only remnants of epithelial cells undergoing apoptosis or necrosis during chronic inflammation associated with chronic injury, or if these cells underwent EMT and entered the bloodstream as intact mesenchymal cells. These assertions, although speculative, are strengthened by the recent report that DCLK1 marks tumor stem cells in *Apc^Min/+^* mice, supporting the functional significance of DCLK1 in neoplasia [15, 16, 25].

This hypothesis is supported by the TCGA analysis of HCC RNA-seq dataset, in which we observed significant upregulation of DCLK1 in HCC tumors compared with adjacent normal tissue (Figure 3A), and increased EMT in DCLK1^High^-expressing HCC tumors (Figure 3B). This study may for the first time provide a rational mechanistic approach to biomarker development and, perhaps, a confirmatory test for cirrhosis.

Prior to malignant transformation, DCLK1 expression in tissue and plasma makes expression sensitive for conversion from normal to pre-neoplasia, considerably limiting its specificity. Nevertheless, DCLK1 expression in cirrhosis may open the doors to evaluating other functional stem cell proteins. Larger, appropriately powered, prospective studies are needed to confirm these hypotheses.

HCC is unique among cancers, occurring mostly in patients with chronic inflammation and cirrhosis [26]. Its treatment is challenging, since HCC is largely refractory to chemotherapy [27]. Thus, advancements in HCC prevention and surveillance may represent the best strategies to reduce the worldwide burden of disease [28]. Our findings suggest that a stem-cell-like cell may play a role in the development of cirrhosis, a key pre-malignant condition and major risk factor for HCC. Such knowledge may help unveil novel targets for chemoprevention and treatment. Identification of early pre-cancerous stem-cell-like markers may aid in the identification of predictive and prognostic biomarkers for HCC [8, 27]. Further studies are needed to more fully identify the importance of DCLK1 in HCC initiation, progression, treatment, and chemoprevention.

Inhibiting liver tumor growth by targeting DCLK1 is important. These new data show the huge potential of siRNA-based therapy. The role of DCLK1 in the regulation of pluripotency in the liver cancer context is novel and may present an exciting new target for anti-cancer therapy. The mechanism of inducing miRNAs may be safe, if recently published reports suggesting that quiescent stem cell populations are dispensable for normal homeostatic processes, but are likely activated during geno/cytotoxic injury and neoplasia, are correct [29, 30]. A recent study also demonstrated that ablation of Dclk1+ cells in *Apc^min/+^* mice resulted in regression of intestinal polyps, without affecting normal intestinal homeostasis [16]. These data provide the rationale for ongoing studies that investigate the role of DCLK1 in the regulation of miRNAs in cancer.

We hypothesize that pluripotency, EMT, cancer stemness, and oncogenesis play a multifaceted role in the initiation, progression, and metastasis of liver cancer. DCLK1 controls these complex cellular signaling pathways, making DCLK1 an attractive candidate or a novel target for treatment of HCC. Furthermore, DCLK1 may also be used as a prognostic biomarker for liver cancer. We plan to pursue these directions in future studies.

## MATERIALS AND METHODS

### Clinical study design

We examined DCLK1 expression in tissue and plasma from patients with HCC. Given the retrospective nature, and the lack of plasma and tissue samples from the same patient, we conducted two separate case-control studies with samples from two repositories. The first study matched archived pathological specimens of HCC to non-HCC controls for the time of diagnosis (year), and evaluated these for DCLK1 expression. The second study compared differences in DCLK1 expression in prospectively obtained, stored, and de-identified plasma from patients with and without HCC.

### Histopathology case-control study

We reviewed the records for all patients who underwent curative resection for liver lesions between June 2000 and December 2010 at the University of Oklahoma Medical Center. HCC inclusion criteria were: (1) no preoperative or pre-biopsy cancer treatment, (2) age 18 years or older, (3) available medical records and clinical data, and (4) surgical tissue/specimen. We excluded patients who only underwent a fine needle biopsy. We selected two cases from each calendar year, except for the last two years, in which three cases were chosen. For each case, we sampled two controls from the same tissue bank: 1) non-cirrhosis controls (NCCs) without liver cirrhosis and without history of HCC, and 2) cirrhosis controls (CCs) with liver cirrhosis, regardless of underlying etiology and without history of HCC. Controls were matched for time of sample collection.

We collected formalin-fixed, paraffin-embedded (FFPE) archived surgical specimen blocks from the patients’ pathology files for analysis. The pathology department then cut two unstained slides for each patient and provided all specimens to the co-investigator (MM). Slides were labeled using a coding system that blinded lab technicians to the source of the slides and the patient's clinical data, and were then stained for DCLK1 testing. Slides were scored by one senior cytopathologist (SAL). Scoring was based on 1) staining intensity and 2) amount of tissue involved. Intensity was measured and scored from 0–3: 0 = no staining, 1 = weak staining, 2 = moderate staining, and 3 = strong staining. The amount of tissue involved was scored from 0–4 based on the percent involvement: no tissue involved = 0%, 1 = < 10% involved, 2 = 10%-40% involved, 3 = 41%-60% involved, and 4 = > 60% involved. Composite scores were generated by multiplying the intensity score by the tissue involvement score (e.g., 3 × 4 = 12). Scoring was done separately for epithelial and stromal tissue. Tissue was considered positive for DCLK1 if the composite multiplied score was ≥ 3.

Twenty-three NCC cases, 22 CCs, and 23 HCCs surgical specimens were included in the histopathology analysis. The histopathologist re-scored them for DCLK1, without looking at the previous scoring, to evaluate for intra-observer agreement. Kappa values over 0.8 are considered excellent agreement; those less than 0.2 are considered very poor. Values of 0.6–0.79 point to good, 0.4–0.59 to moderate, and 0.2–0.39 to weak agreement.

Medical records of all patients with an HCC diagnosis were then reviewed. Demographic information (age, gender, race) and clinical and pathological characteristics, such as sites of primary mass, stage at time of diagnosis (TNM staging; The American Joint Committee on Cancer [AJCC]), pathological classifications (liver cell carcinoma, fibrolamellar, cholangiocarcinoma, or mixed), tumor grade (the degree of differentiation), severity of the underlying liver disease (Child-Pugh classification), performance status as measured by the Eastern Cooperative Oncology group (ECOG), first alpha fetoprotein (AFP), and vascular and lymph invasion on tissue biopsy were recorded.

### Plasma study

Plasma samples from patients with and without HCC were stored at less than −80°C and were examined for DCLK1 expression. Thirty-three patients, 18 HCC and 15 CC, were prospectively recruited, and their blood samples were withdrawn from storage. Inclusion criteria were: patients with elevated γ-Glutamyl transpeptidase (GGT), AFP, and confirmed due to cirrhosis or HCC (based on biopsied). Exclusion criteria were: 1) age less than 18 years or 2) pregnancy. Subjects were categorized into two groups: 1) patients with elevated GGT due to HCC, and 2) patients with elevated GGT from cirrhosis, who served as cirrhotic controls. Finally, plasma from 8 healthy volunteers was used as our normal non-cirrhosis control (NCCs). All plasma samples were de-identified, and were used for protein analysis. Technicians performing and analyzing the western blots were blinded to case control status.

### Western blot analysis

Plasma samples were purified using a protein depletion kit purchased from Norgen, Inc. (ProteoSpin Abundant Serum Protein Depletion Kit). Samples were separated on a 10% SDS-PAGE gel and were transferred to an Immobilon membrane. Following blocking, the membrane was probed overnight with DCLK1 primary antibody (Abcam, 1:1000). The membrane was subsequently probed with secondary antibody conjugated with horseradish peroxide for 1 h. The 82-kDa DCLK1 protein was detected using ECL™ Western Blotting detection reagents (Amersham-Pharmacia).

### ELISA

The plasma DCLK1 level was quantified using a commercially available ELISA assay (USCN Life Science, Inc., Wuhan, China). The 96-well plate coated with monoclonal antibody against DCLK1 was pre-blocked. Different concentrations (0–10 ng/ml) of purified DCLK1 protein were used to create a standard curve. Serum samples were diluted 1:4 and 1:10 with PBS. The diluted serum samples and purified DCLK1 proteins were added into the plate and incubated for 2 h at room temperature. The plate was then incubated with biotinylated polyclonal antibody against DCLK1 for 1 h at room temperature. After three washes, the plate was incubated with Streptavidin conjugated with horseradish peroxidase (HRP) for 30 min at room temperature. Finally, the plate was developed with HRP substrate for 20 min and terminated by adding stop solution. The value of OD 450 nm was measured using a microplate reader. The concentration of DCLK1 in serum samples was determined based on the standard curve constructed using purified DCLK1.

### Immunohistochemistry

Heat-induced epitope retrieval was performed on formalin-fixed paraffin-embedded sections by utilizing a pressurized decloaking chamber (Biocare Medical LLC, Concord, CA) in citrate buffer (pH 6.0) at 99°C for 18 min. Brightfield: slides were incubated in 3% hydrogen peroxide at room temperature for 10 min. After incubation with primary antibody (DCLK1 1:100 [rabbit], Abcam, Cambridge, MA) overnight at 4^°^C, the slides were incubated in a Promark peroxidase-conjugated polymer detection system (Biocare Medical, LLC) for 30 min at room temperature. After washing, slides were developed with diaminobenzidine (Sigma, St. Louis, MO). Slides were examined on a Nikon Eclipse Ti motorized microscope paired with the DS-Fi2 color and CoolSnap ES2 monochrome digital cameras utilizing DIC enhanced PlanApo objectives operated by the NIS-Elements Microscope Imaging Software platform (Nikon Instruments, Melville, NY). Immunohistochemically stained slides were read by one investigator (SL).

### Statistical analysis

For the histopathology study analyses, we compared the mean epithelial or stromal scores of DCLK1 using ANOVA and Tukey's-adjusted multiple comparisons. For the plasma study, the proportions of samples with Western blot analysis positive for DCLK1 were compared using χ2 or Fisher's exact test, as appropriate, with Bonferroni's-adjusted pair-wise comparisons. Bonferroni's-adjusted *p*-values are presented and were compared to a 2-sided 0.05 alpha level to determine statistical significance. Data were presented as either number (%) or mean ± *SD*. Intra-observer variability of DCLK1 scoring was quantified using simple Kappa statistics. Multiple clinical variables among patients with HCC were examined in relation to DCLK1 composite scores, using either *χ2* or Fisher's exact test, as appropriate, for categorical variables, or Wilcoxon rank sum test for continuous variables.

### Analysis of NCBI GEO and TCGA patient data

The Cancer Genome Atlas’ (TCGA) Liver Hepatocellular Carcinoma (LIHC) dataset [31] was downloaded from the University of California Santa Cruz (UCSC) genome browser and sorted using R (v3.2.0). The data set had analysis obtained from 50 normal and 371 HCC samples. DCLK1 expressing tissues was classified based on the expression levels of DCLK1 as DCLK1^Low^- (0–25 percentile, *n* = 93), DCLK1^Mid^- (25–75 percentile, *n* = 185) and DCLK1^High^- (75–100 percentile, *n* = 93) [32]. *Statistical Analyses*. Statistical analyses were performed in GraphpadPrism 6.0. For non-parametric data the Mann-Whitney *U* test was used.

### Real-time reverse transcription-polymerase chain reaction analyses

Total RNA isolated from tumor xenografts was subjected to reverse transcription using Superscript™ II RNase H-Reverse Transcriptase and random hexanucleotide primers (Invitrogen, Carlsbad, CA). The complementary DNA (cDNA) was subsequently used to perform real-time (RT) polymerase chain reaction (PCR) by SYBR™ chemistry (SYBR Green I, Molecular Probes, Eugene, OR) for specific transcripts using gene-specific primers and JumpStart™ Taq DNA polymerase (Sigma-Aldrich). The crossing threshold value was noted for the transcripts and normalized with β-actin messenger RNA (mRNA). The quantitative changes in mRNA were expressed as fold-change relative to control with ± *SEM* value. The following primers were used:

**Table:** 

β-actin:	forward: 5′-GGTGATCCACATCTGCTGGAA-3′,
	reverse: 5′-ATCATTGCTCCTCCTCAGGG-3′;
DCLK1:	forward: 5′- CAGCAACCAGGAATGTATTGGA-3′,
	reverse: 5′- CTCAACTCGGAATCGGAAGACT-3′;
cMYC:	forward: 5′-CACACATCAGCACAACTACGCA-3′,
	reverse: 5′-TTGACCCTCTTGGCAGCAG-3′;
ZEB1:	forward: 5′-AAGAATTCACAGTGGAGAGAAGCCA-3′,
	reverse: 5′-CGTTTCTTGCAGTTTGGGCATT-3′;
ZEB2:	forward: 5′-AGCCGATCATGGCGGATGGC-3′,
	reverse: 5′-TTCCTCCTGCTGGGATTGGCTTG-3′;
SNAIL:	forward: 5′-AAGGCCTTCTCTAGGCCCT-3′,
	reverse: 5′-CGCAGGTTGGAGCGGTCAG-3′;
SLUG:	forward: 5′-TGCTTCAAGGACACATTA-3′,
	reverse: 5′-CAGTGGTATTTCTTTAC-3′;
NANOG:	forward: 5′-ACCAGAACTGTGTTCTCTTCCACC-3′,
	reverse: 5′-CCATTGCTATTCTTCGGCCAGTTG-3′;
KLF4:	forward: 5′-CCAATTACCCATCCTTCCTG-3′,
	reverse: 5′-CGATCGTCTTCCCCTCTTTG-3′;
OCT4:	forward: 5′-AAGCGATCAAGCAGCGACTAT-3′,
	reverse: 5′-GGAAAGGGACCGAGGAGTACA-3′;
LIN28B:	forward: 5′-GATGTATTTGTACACCAA-3′
	reverse: 5′-TACCCGTATTGACTCAAGGCC-5′

### miRNA analysis

Total RNA isolated from tumor xenografts and cancer cells was subjected to reverse transcription with Superscript II RNase H-Reverse Transcriptase and random hexanucleotide primers (Invitrogen). The cDNA was subsequently used to perform RT-PCR by SYBR chemistry for *pri-let-7a, pri-miR-144, pri-miR-200a, pri-miR-143*, and *pri-miR-145* transcripts using specific primers and JumpStart Taq DNA polymerase. The crossing threshold value was noted for *pri-let-7a, pri-miR-144, pri-miR-143, pri-miR-145*, and *pri-miR-200a* miRNAs, and normalized with *U6* pri-miRNA. The changes in pri-miRNAs were expressed as fold-change relative to control ± *SEM* values. [22] The following primers were used:

**Table:** 

*pri-U6*:	forward: 5′-CTCGCTTCGGCAGCACA-3′,
	reverse: 5′-AACGCTTCACGAATTTGCGT-3′;
*pri-let-7a*:	forward: 5′-GAGGTAGTAGGTTGTATAGTTTAGAA-3′,
	reverse: 5′-AAAGCTAGGAGGCTGTACA-3′;
*pri-miR-200a*:	forward: 5′-TTCCACAGCAGCCCCTG-3′,
	reverse: 5′-GATGTGCCTCGGTGGTGT-3′.
*pri-miR-143*:	forward: 5′-AGGGCCAGCAGCAGGC-3′,
	reverse: 5′-TCAGGAAATGTCTCTGGCTGTG-3′.
*pri-miR-145*:	forward: 5′-GGATGCAGAAGAGAACTCCA-3′,
	reverse: 5′-CCTCATCCTGTGAGCCAG-3′.

### Luciferase reporter gene assay

Huh7.5 cells were transfected with a plasmid containing the firefly luciferase (*Photinus pyralis*) gene with a complementary *miR-143/145* and *let-7a* (separate plasmids) binding site at its 3′ UTR (Signosis, Inc., Sunnyvale, CA). The cells were also co-transfected with the *Renilla* luciferase-expressing plasmid pRL-TK (Promega) as an internal control.

Following transfection, the cells were treated with NPs, NP-siSCR, or NP-siDCLK1, and were subjected to luciferase activity measurement. Luciferase activity was determined per the manufacturer's instructions (Dual-Luciferase Reporter Assay System; Promega) using a Biotek Synergy HT multi plate reader (BioTek, Winooski, VT) as described previously [17, 22].

Plasmids containing binding sites for *miR-200a, miR-200b*, and *miR-200c* at the 3′UTR of the firefly luciferase gene were obtained from Switchgear Genomics (Menlo Park, CA). Huh7.5 cells were transfected with the abovementioned plasmids, along with pRL-TK. Following transfection, the cells were treated with NPs, NP-siSCR, or NP-siDCLK1 and were subjected to luciferase activity measurement using a Biotek Synergy HT multi plate reader per the manufacturer's instructions.

The activity, normalized to *Renilla* luciferase activity, is presented as relative luciferase units relative to control ± *SEM* values. Assays were performed in triplicate wells. Experiments were repeated three times.

### Small interfering RNAs

We obtained a DCLK1 siRNA (siDCLK1) sequence targeting the coding region of DCLK1 (accession No. NM_004734; GGGAGUGAGAACAAUCUACtt) and scrambled siRNAs (si-SCR) not matching any of the human genes, from Ambion, Inc. (Austin, TX).

### Synthesis and characterization of DCLK1 siRNA nanoparticles

Poly(lactide-*co*-glycolide) acid nanoparticles (PLGA NPs) were synthesized using a double emulsion solvent evaporation technique as described earlier [19, 33]. Briefly, siRNA (DCLK1 or scrambled) was condensed on the cationic polymer poly(ethyleneimine), PEI, to form an siRNA-PEI complex. This complex was added to PLGA in chloroform, vortexed, and transferred to 2% polyvinyl alcohol. This emulsion was sonicated and allowed to evaporate overnight. The size, polydispersity index, and zeta-potential measurements of synthesized siRNA NPs were determined using diffraction light scattering (DLS) utilizing Zeta PALS (Brookhaven Instruments, Holtsville, NY).

### Xenograft tumor model

Athymic nude mice (Jackson Laboratory, Bar Harbor, Maine) were housed in pathogen-free conditions and cared for in accordance with guidelines set forth by the American Association for Accreditation of Laboratory Animal Care and the U.S. Public Health Service Commissioned Corps’ “Policy on Human Care and Use of Laboratory Animals.” All studies were approved and supervised by the University of Oklahoma Health Sciences Center's Institutional Animal Care and Use Committee (IACUC). To generate tumor xenografts, Huh7.5 cells (4 × 10^6^) (50 μL) were subcutaneously injected into the flanks of 4-to-6-week-old mice (*n* = 4 animals per group). Tumors were measured using a caliper and the volume was calculated as (length × width^2^) × 0.5. The tumors were palpable 18 days after injection. NPs were reconstituted in sterile normal saline and injected *via* i.p. Each tumor-bearing animal was injected on days 18, 21, 24, 27, and 30, with one of the following: 50 μl (5 μM) of siRNA-NP preparation NP alone (Control), NP-siScrambled (NPsiSCR), or NPsiDCLK1. All mice were killed on day 31 (Figure 4A) and the tumors were excised.

## SUPPLEMENTARY TABLE


