# Supplementary material for: Plasma DCLK1 is a marker of hepatocellular carcinoma (HCC): Targeting DCLK1 prevents HCC tumor xenograft growth via a microRNA-dependent mechanism
Source: Oncotarget. 2015 Oct 16;6(35):37200–15. doi: 10.18632/oncotarget.5808 (PMC4741924; doi:10.18632/oncotarget.5808)
Supplement: Supplementary file 1 [file oncotarget-06-37200-s001.pdf]

SUPPLEMENTARY TABLE

Supplementary Table S1: Comparison between DCLK-1 positive and negative HCC patients

	DCLK-1 (+) *N* = 19	DCLK-1 (-) *N* = 4	*P* value
Age, mean (±SD),y	61.3 ± 14.3	63.5± 12.7	0.78
Men, no. (%)	11/19 (58)	2/4 (50)	0.77
AFP (Median)	166.9	5.5	0.07
White race, no. (%)	10/19 (53)	3/4 (75)	0.41
Viral etiology, no. (%)	12/19 (63)	2/4 (50)	1.00
Child C, no. (%)	4/17 (24)	0/3 (0)	1.00
Advanced stage (III or IV), no. (%)	16/18 (79)	2/4 (50)	0.27
Size of the largest lesion, mean (±SD), cm	8.6 ± 5.6	7.3 ± 6.7	0.73
Presence of metastasis, no. (%)	8/19 (42)	1/3 (33)	0.77
Presence of lymphatic invasion, no. (%)	9/19 (47)	2/3 (67)	0.53
Presence of vascular invasion, no. (%)	5/18 (28)	0/4 (0)	0.54
Presence of multiple lesions, no. (%)	14/19 (74)	1/4 (25)	0.06

Abbreviation: DCLK-1: doublecortin-like kinase-1; HCC: hepatocellular carcinoma.
